# Efficacy of Mammographic Artificial Intelligence-Based Computer-Aided Detection in Predicting Pathologic Complete Response to Neoadjuvant Chemotherapy

**DOI:** 10.3390/life14111449

**Published:** 2024-11-08

**Authors:** Ga Eun Park, Bong Joo Kang, Sung Hun Kim, Han Song Mun

**Affiliations:** Department of Radiology, Seoul Saint Mary’s Hospital, College of Medicine, The Catholic University of Korea, Seoul 06591, Republic of Korea

**Keywords:** breast cancer, artificial intelligence, computer-aided diagnosis, mammography, neoadjuvant chemotherapy

## Abstract

This study evaluates the potential of an AI-based computer-aided detection (AI-CAD) system in digital mammography for predicting pathologic complete response (pCR) in breast cancer patients after neoadjuvant chemotherapy (NAC). A retrospective analysis of 132 patients who underwent NAC and surgery between January 2020 and December 2022 was performed. Pre- and post-NAC mammograms were analyzed using conventional CAD and AI-CAD systems, with negative exams defined by the absence of marked abnormalities. Two radiologists reviewed mammography, ultrasound, MRI, and diffusion-weighted imaging (DWI). Concordance rates between CAD and AI-CAD were calculated, and the diagnostic performance, including the area under the receiver operating characteristics curve (AUC), was assessed. The pre-NAC concordance rates were 90.9% for CAD and 97% for AI-CAD, while post-NAC rates were 88.6% for CAD and 89.4% for AI-CAD. The MRI had the highest diagnostic performance for pCR prediction, with AI-CAD performing comparably to other modalities. Univariate analysis identified significant predictors of pCR, including AI-CAD, mammography, ultrasound, MRI, histologic grade, ER, PR, HER2, and Ki-67. In multivariable analysis, negative MRI, histologic grade 3, and HER2 positivity remained significant predictors. In conclusion, this study demonstrates that AI-CAD in digital mammography shows the potential to examine the pCR of breast cancer patients following NAC.

## 1. Introduction

Neoadjuvant chemotherapy (NAC) is a well-established management method for breast cancers, significantly reducing the need for mastectomy and axillary lymph node dissection without increasing the risk of loco-regional recurrence [1]. Moreover, achieving a pathologic complete response (pCR) serves as a surrogate marker for improved survival outcomes [1,2,3]. Accurate assessment of residual tumors after NAC is imperative for determining the requisite extent of surgery and for monitoring the effectiveness of the treatment [4]. Numerous studies have confirmed that an MRI is the most accurate imaging modality for predicting residual tumors after NAC, outperforming conventional methods such as clinical breast examination, mammography, and ultrasound [5,6,7]. Additionally, the effectiveness of MRI post-NAC has been demonstrated to vary depending on tumor subtype, histologic grade, and morphologic patterns observed on an MRI [8,9,10,11].

Mammography remains a widely used tool for breast cancer screening worldwide and has contributed effectively to reducing mortality among breast cancer patients [12,13]. Despite its widespread use, mammography’s inherent limitations result in sensitivity rates ranging from 70 to 90%. Traditional computer-aided detection (CAD) systems, designed to aid mammographic interpretation and mitigate these limitations, have historically displayed low specificity and a high recall rate due to numerous false positives, leading to minimal improvements in performance or cost-effectiveness [14,15,16]. 

With advancements in deep learning and artificial intelligence (AI), a new generation of computer-assisted image analysis tools has emerged. Various AI-based CAD systems for mammography are now commercially available to assist in breast cancer detection and diagnosis. While diagnostic performance varies across studies, AI-CAD has shown non-inferior or even superior performance compared to radiologists, establishing its effectiveness as a reliable decision-support tool [17,18,19,20,21,22,23,24]. It has demonstrated a reasonable detection rate even in dense breasts, a well-known factor that reduces the sensitivity of mammography [20,25]. There are also studies that have utilized mammography AI-CAD in diverse ways. Some have explored replacing one of the radiologists in double reading systems with AI-CAD [26,27], while others have aimed to reduce the workload by triaging cases, thus improving the workflow for mammography interpretation [19,28]. Recently, studies have even shown that AI-CAD can be utilized to predict future breast cancer based on its abnormality score [29]. Additional research has used AI-CAD to reduce recall rates [30,31] and to analyze calcifications on mammography [32,33].

In our readings of mammograms for breast cancer patients undergoing neoadjuvant chemotherapy, we observed significant differences in AI-CAD results based on treatment response. Building on this observation, this study aims to evaluate AI-CAD’s performance in predicting pCR and to compare it with other imaging modalities including conventional CAD, ultrasound, MRI, and DWI.

## 2. Materials and Methods

### 2.1. Study Population

This study received approval from the Institutional Review Board (IRB). Due to the retrospective nature of the study, the requirement for patient consent was waived. Between January 2020 and December 2022, 175 patients diagnosed with invasive breast cancer, who completed neoadjuvant chemotherapy (NAC) and underwent surgery, were included in this study. These patients had both their initial (pre-NAC) and preoperative (post-NAC) mammography analyzed using conventional CAD and AI-CAD. Patients with progressive disease (*n* = 7), stage IV cancer (*n* = 5), those who did not undergo surgery at our institution after neoadjuvant chemotherapy (*n* = 4), and those with bilateral malignancy (*n* = 3) were excluded. Additionally, cases where either pre- or post-NAC mammography, ultrasound, or MRI images were unavailable (*n* = 21), and cases where AI-CAD or conventional CAD analysis failed (*n* = 3) were also excluded. In the end, 132 patients were included in this study.

### 2.2. Image Interpretation

Image interpretation was retrospectively performed by two dedicated breast imaging radiologists with 20 and 18 years of experience with consensus. Findings were evaluated for mammography and categorized into mass, calcifications, and mass with calcifications. For ultrasound and MRI, the categories were mass, nonmass enhancement, and mass with nonmass enhancement. Lesion distribution for MRI was assessed as single, multifocal, multicentric, diffuse/inflammatory, and bilateral. Lesion size for mammography, ultrasound, and MRI was measured as the maximal diameter for both pre- and post-NAC. For multiple clearly separated tumors, the diameters were summed. If a uniquely high signal lesion on a high b-value (1000 s/mm^2^) DWI was detected, the ADC value was measured by drawing an ROI at the representative area. Negative exams were defined as follows, with all other cases considered positive: a size measurement of 0 on mammography, ultrasound, or MRI; no markings on CAD or AI-CAD; and an ADC measurement of 0 or greater than 1300 (×10⁻⁶ mm^2^/s) in DWI [34].

### 2.3. Conventional CAD and AI-CAD Analysis

Two commercially available CAD systems were tested: the conventional R2 ImageChecker Cenova (version 1.0, Hologic Inc., Bedford, MA, USA) and the AI-powered Lunit INSIGHT for Mammography (v1.1.4.3, Lunit Inc., Seoul, Republic of Korea, available at https://insight.lunit.io, accessed on 7 November 2024). These systems are designed for the detection and diagnosis of breast cancer in digital mammography and are automatically applied through post-processing after the mammography examination. Conventional CAD presented three kinds of markers according to mammography findings on each view of mammograms: a triangle (▲) for calcifications, an asterisk (*) for mass/asymmetry, and a cross (+) for combined mass with calcifications. 

The AI-CAD used in this study is a commercial software developed to assist in breast cancer detection, with regulatory approval in Europe (Conformité Européenne (CE) marked) and the US (cleared by the Food and Drug Administration). According to the vendor, this AI-CAD was developed using deep convolutional neural networks (CNNs) and was trained on approximately 170,000 mammograms from a multinational mammogram dataset [20]. When detecting suspicious findings, the AI-CAD visually marks the area with lines or colors and provides an abnormality score for the region. The abnormality score is provided in a percentage range of 0–100%, with higher values indicating an increased likelihood of cancer presence on the mammogram. This AI-CAD software (v1.1.4.3) presents its results as separate grayscale images that include an overall per-breast abnormality score for each CC (craniocaudal) and MLO (mediolateral oblique) image, making the AI-CAD algorithm output more interpretable and transparent.

Mammographic breast density provided by AI-CAD is classified into four categories, almost entirely fatty, scattered, heterogeneous, and extremely dense according to the *Breast Imaging Reporting and Data System (BI-RADS) 5th edition* [35].

### 2.4. Medical Record and Histopathological Reviews

The patient’s age at the time of the mammogram and operation type were retrieved from medical records. Pathological reports from biopsy specimens before NAC were meticulously reviewed to ascertain various tumor characteristics, such as histological grade, presence of lymph node involvement, estrogen receptor (ER), progesterone receptor (PR), human epidermal growth factor receptor (HER)2, and Ki-67 status. A cut-off value of ≥1% was used for ER and PR positivity. HER2 expression was semi-quantitatively assessed with scores of 0, 1, 2, or 3; a score of 3 indicated HER2 positivity, while scores of 0 or 1 suggested HER2 negativity. Tumors with a HER2 score of 2 required gene amplification testing to determine HER2 status. Positive Ki-67 expression was recognized when ≥20% of cancer cell nuclei were stained. IHC subtypes were formulated according to the 2013 St. Gallen International Breast Cancer Conference guidelines, categorizing them as (1) luminal A (ER or PR+, HER2-, and low Ki-67), (2) luminal B (ER or PR+, HER2+, and/or high Ki-67), (3) HER2-enriched (ER-, PR-, and HER2+), and (4) triple-negative (ER-, PR-, and HER2-). Final pathologic outcomes were based on surgical results and then divided into pCR and non-pCR. In this study, pCR was defined as neither invasive cancer nor DCIS within the breast. In addition, the size of the residual tumor (invasive cancer and ductal carcinoma in situ (DCIS)) in surgical specimens was recorded.

### 2.5. Statistical Analysis

We compared pCR and non-pCR group differences for categorical variables using the chi-squared test or Fisher’s exact test as appropriate, and for continuous variables, differences were compared using Student’s *t*-test or the Wilcoxon signed-rank test depending on their distribution. The concordance rate (%) between mammography and AI-CAD, and CAD in post-NAC, were evaluated. We evaluated the association between pCR and imaging modalities in post-NAC such as CAD, AI-CAD, mammography, ultrasound, MRI, and DWI. Then, we compared the diagnostic performance for each imaging modality of pCR prediction using sensitivity, specificity, positive predictive value (PPV), negative predictive value (NPV), and area under the receiver operating characteristics curve (AUC). We conducted univariate logistic regression analyses to explore the association between potential predictors for pCR, selecting variables with *p*-values < 0.05. These variables were then included in a multivariable logistic regression model using a stepwise selection process. A *p*-value < 0.05 was designated as indicative of statistical significance. All statistical analyses were conducted using SAS 9.4 (SAS Institute, Cary, NC, USA).

## 3. Results

Table 1 presents the baseline characteristics of the study population. The study included 132 patients with an average age of 51.6 ± 10.2 years range (23–77 years), and there was no significant age difference between the pCR and non-pCR groups. Image findings, lesion size on pre-NAC mammography, ultrasound, MRI, and the mean ADC values showed no significant differences between the pCR and non-pCR groups. Unlike ultrasound and MRI, mammography did not show a significant size difference between pre-NAC and post-NAC. Histologically, the pCR group exhibited a higher proportion of grade 3 tumors (62.2% vs. 29.5%, *p* = 0.001) and lower lymph node involvement compared to the non-pCR group (21.6% vs. 40.0%, *p* = 0.047). There were significant associations between pCR and negative ER and PR status, as well as positive HER2 status and higher Ki-67 levels. Additionally, significant variations were also observed in the distribution of molecular subtypes and pCR rates, with the HER2-enriched subtype showing the highest pCR rate (20/30, 66.7%) and the luminal A subtype showing the lowest (1/19, 5.3%). Most patients (87.1%) underwent breast-conserving surgery.

Table 2 revealed the concordance between mammography, AI-CAD, and CAD according to mammography findings. Overall, AI-CAD demonstrated slightly higher concordance with mammography compared to CAD in both pre-NAC (97.0% vs. 90.9%) and post-NAC mammography (89.4% vs. 88.6%). When analyzed by type, AI-CAD showed a higher concordance rate than CAD in mass detection in pre-NAC mammography (96.9% vs. 81.3%), while CAD displayed 100% concordance for mass with calcifications. Both CAD systems showed 100% concordance in cases of calcifications. In post-NAC mammography, no difference was observed between the two systems in the detection of mass with calcifications or mass. However, AI-CAD achieved 100% concordance in negative cases, while CAD maintained 100% concordance in calcifications. The median abnormality score for AI-CAD significantly decreased from 96 [77.25, 99] in pre-NAC to 42.5 [0, 91] in post-NAC. Excluding negative findings, the abnormality score for mass-type lesions dropped markedly from 90 [62.75, 97] to 45.5 [14.5, 87.5].

**Figure 1:**
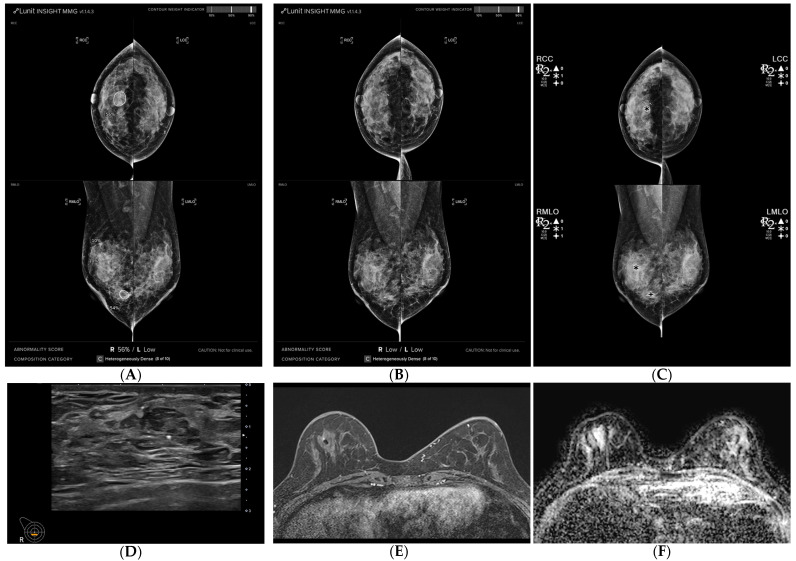
A 54-year-old woman diagnosed with breast cancer presenting as a mass with calcifications, exhibiting a surgically confirmed pathologic complete response (pCR) following neoadjuvant chemotherapy (NAC). (**A**,**B**) Mammography was analyzed using AI-CAD, identifying an area in the left central breast of a mass with calcifications in pre-NAC images (AI-CAD score 98% on the MLO view), and no mark was detected in post-NAC images. (**C**) Utilizing conventional CAD, identifying an area in the left central breast corresponding to the calcifications in post-NAC images. (**D**) Post-NAC ultrasound recorded a reduction in mass size from 26 mm. (**E**) Post-NAC maximal intensity projection (MIP) of contrast-enhanced MRI (CE-MRI) documented a mass reduction from 36 mm. (**F**) Post-NAC ADC map of DWI displayed regression of the mass.

**Figure 2:**
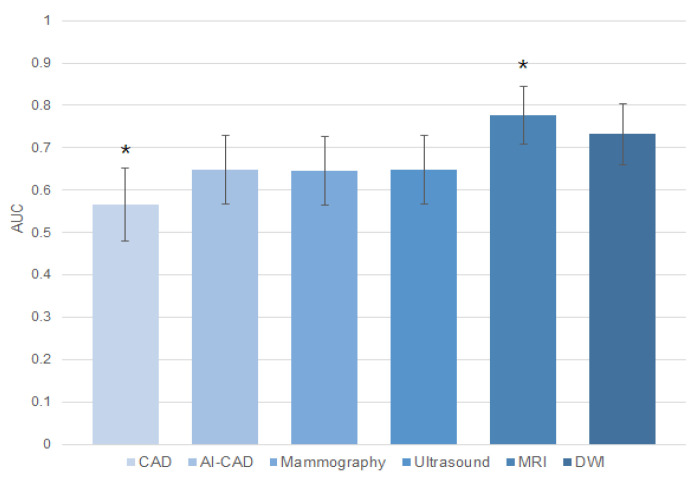
Area under the ROC curves for pCR prediction in post-NAC imaging modalities. * denotes a statistically significant difference.

Table 3 presents the diagnostic performance of pCR prediction across imaging modalities in post-NAC. CAD demonstrated a sensitivity of 77.9%, a specificity of 35.1%, a PPV of 75.5%, and an NPV of 38.2% for pCR prediction. AI-CAD demonstrated a sensitivity of 72.6%, a specificity of 56.8%, a PPV of 81.2%, and an NPV of 44.7%. Mammography yielded a sensitivity of 83.2%, a specificity of 45.9%, a PPV of 79.8%, and an NPV of 51.5%. Ultrasound revealed similar results with a sensitivity of 86.3% and a specificity of 43.2%, with a PPV of 79.6% and an NPV of 55.22%. MRI exhibited a sensitivity of 76.8% and a specificity of 78.4%, along with a PPV of 90.1% and an NPV of 56.9%. DWI showed the highest specificity and a PPV at 100% but reported the lowest sensitivity at 46.3%, with an NPV of 42.1%. Specificity was highest in DWI (100%), followed by MRI (78.4%), AI-CAD (56.8%), mammography (45.9%), ultrasound (43.2%), and CAD (35.1%) (Figure 1). The AUC was highest for MRI at 0.776 [95% CI: 0.695, 0.844] and lowest for CAD at 0.565 [0.476, 0.651]. Comparison of AUCs across imaging modalities revealed that MRI and CAD showed significant differences compared to all other modalities (Figure 2). The AUC for AI-CAD was 0.647 [0.559, 0.728], with no statistically significant differences compared to mammography, ultrasound, or DWI (*p* > 0.05).

Table 4 presents the association between pCR and imaging modalities in post-NAC. CAD demonstrated no significant association with pCR (*p* = 0.124), with 64.9% of the pCR group testing positive versus 77.9% of the non-pCR group. Conversely, AI-CAD was significantly associated with pCR (*p* = 0.002), with 56.8% of the pCR group testing negative compared to 27.4% of the non-pCR group. In addition, the AI-CAD’s abnormality score of AI-CAD was significantly lower in the pCR group (28.8 ± 38.3) than in the non-pCR group (52.7 ± 41.4, *p* = 0.001). Mammography was a significant differentiator between pCR and non-pCR (*p* = 0.001), with 45.9% of the pCR group testing negative compared to 16.8% of the non-pCR group. Similarly, ultrasound demonstrated a significant difference (*p* < 0.001), with 43.2% of the pCR group testing negative compared to 13.7% of the non-pCR group. MRI showed a strong association (*p* < 0.001), with 78.4% of the pCR group testing negative versus 23.2% of the non-pCR group. DWI also demonstrated a strong association (*p* < 0.001), as all pCR groups tested negative compared to 47.4% of the non-pCR group.

Table 5 presents the results of univariate and multivariable logistic regression analysis for pCR prediction. In the univariate analysis, negative AI-CAD, mammography, ultrasound, and MRI were significantly associated with higher odds of pCR (OR = 3.48, 95% CI: 1.58, 7.69, *p* = 0.002; OR = 4.20, 95% CI: 1.81, 9.73, *p* = 0.001; OR = 4.81, 95% CI: 2.00, 11.5, *p* < 0.001; OR = 12.0, 95% CI: 4.81, 30.1, *p* < 0.001, respectively). Histologic grade 3 was significantly associated with higher odds of pCR (OR = 3.93, 95% CI: 1.77, 3.73, *p* = 0.01). ER and PR positivity strongly predicted pCR (OR = 0.33, 95% CI: 0.15, 0.74, *p* = 0.007; OR = 0.33, 95% CI: 0.14, 0.81, *p* = 0.015). HER2 positivity and higher levels of Ki-67 strongly predicted pCR (OR = 7.35, 95% CI: 2.92–18.5, *p* < 0.001; OR = 2.99, 95% CI: 1.19–7.48, *p* = 0.02). 

In the multivariable analysis, negative MRI remained a significant predictor (adjusted OR = 12.6, 95% CI: 3.28, 48.5, *p* < 0.001). Histologic grade 3 and HER2 positivity also remained significant predictors (adjusted OR = 9.03, 95% CI: 2.40, 33.9, *p* = 0.001; adjusted OR = 14.68, 95% CI: 3.42, 62.9, *p* < 0.001).

## 4. Discussion

To the best of our knowledge, this is the first study to demonstrate the potential of a commercial AI-CAD system for digital mammography to predict the pCR of breast cancer patients undergoing NAC. In this study, AI-CAD showed better performance in predicting pCR on post-NAC mammography than conventional CAD. While it did not reach the level of an MRI, known as the best modality, its performance was comparable to that of mammography, ultrasound, and DWI.

A study conducted in 2022 [36] developed a deep learning model using digital mammography to predict pCR. This model was trained and validated on pre-NAC mammograms from 400 patients and tested on a set of 53 patients. The AUC of this model was 0.71 (0.53–0.90), with a sensitivity of 76% at a fixed specificity of 90%. In our study, the AUC of AI-CAD was 0.65 (0.56–0.73), with a sensitivity of 72.6% and a specificity of 56.8%. Given that this commercial AI-CAD was developed to assist in breast cancer detection and diagnosis on mammography, rather than being trained to predict pCR, these results can be considered satisfactory. Additionally, AI-CAD has the advantage of marking the location of suspicious findings on mammography alongside the abnormality score, allowing readers to easily understand the basis of its assessment. These results were likely possible because the progressive reduction in malignancy-related suspicious findings during NAC was reflected in the AI-CAD.

We evaluated the concordance between conventional CAD and AI-CAD systems in pre- and post-NAC mammography. AI-CAD demonstrated a higher concordance rate with mammography compared to CAD in both pre- and post-NAC settings. Specifically, the concordance rate for AI-CAD was 97.0% in pre-NAC and 89.4% in post-NAC, while for CAD, it was 90.9% in pre-NAC and 88.6% in post-NAC. Notably, the difference between the two systems was particularly pronounced in the detection of mass type in pre-NAC mammography, and AI-CAD showed superior performance (96.9% vs. 81.3%). In contrast, CAD misclassified some of these cases as false negatives. This finding highlights AI-CAD’s potential in improving diagnostic accuracy in breast cancer imaging. In post-NAC mammography, AI-CAD demonstrated strong concordance in detecting negative findings with a 100% concordance rate, whereas CAD had a lower concordance rate of 78.8%, as it often marked false positive findings, leading them to be classified as positive exams. An additional interesting finding was that all patients in this study who underwent NAC had a marker clip placed in the main malignancy. Upon analyzing the results, we confirmed that the placement of the marker clip during NAC did not affect the performance of CAD or AI-CAD in their assessments.

The concept of pathologic complete response (pCR) remains a subject of debate, particularly regarding its definition, evaluation methods, and predictive value for patient outcomes. From a locational perspective, various definitions of pCR are documented in the literature, often focusing on either the breast alone or on both the breast and axillary lymph nodes to assess the pathologic response [37]. From the perspective of residual tumor burden, some studies define pathologic response criteria based on the presence or absence of residual disease (RD). pCR is characterized by the complete absence of RD, while RD itself encompasses a wide range of conditions, from near-pCR to minimal tumor reduction. Residual in situ (RIS) refers to the presence of isolated non-invasive tumors, while residual invasive carcinoma (RIC) involves histologic evidence of invasive disease in patients who have shown a complete clinical response [38]. In a clinical setting, the size of the invasive tumor after NAC is what determines the ypT stage, which serves as the key metric for assessing the oncologic response to NAC. Although the presence of in situ components does not influence the ypT stage or response assessment, complete excision of these components following NAC is necessary to achieve negative surgical margins and reduce the risk of local recurrence [39]. Von Minckwitz et al. [40] have shown that patients with residual in situ carcinoma after NAC have a higher risk of relapse compared to those with no remaining tumors in the breast or lymph nodes. The most recent international consensus conference on NAC in primary breast cancer defined pCR as the absence of invasive cancer in both the breast and lymph nodes, with the stipulation that DCIS should be analyzed separately [38].

In this study, pCR was assessed only in the breast, as mammography is not optimal for accurately evaluating axillary lymph nodes. We defined pCR as the complete absence of residual disease, which includes no invasive cancer of in situ lesions. The approach was necessary because, after NAC, some cases showed a response on MRI but still had residual calcifications visible on mammography. In this study, 19% (25/132) of the post-NAC mammography had residual calcifications. These cases included both those with calcifications only on pre-NAC mammography (*n* = 3) and cases of the mass with calcifications on pre-NAC mammography, where the mass regressed post-NAC, leaving only calcifications (*n* = 22). When assessing these residual calcifications, CAD indiscriminately marked all 25 cases. In contrast, AI-CAD evaluated the suspiciousness of the calcifications, classifying cases with a probability of malignancy below 10% as negative exams. AI-CAD identified 6 of the 25 cases as negative, 4 of which were pCR cases.

This study highlights the strengths and limitations of different imaging modalities in assessing pCR. MRI showed high specificity (78.4%) and PPV (90.1%), making it a reliable tool for differentiating pCR from non-pCR. While DWI had perfect specificity (100%) and PPV (100%), its sensitivity was lower (46.3%), limiting its overall utility. Mammography and ultrasound had comparable sensitivities (83.2% and 86.3%, respectively), but their specificities were lower (45.9% and 43.2%). AI-CAD, though slightly less sensitive than CAD (72.6% vs. 77.9%), outperformed CAD in terms of specificity (56.8% vs. 35.1%), PPV (81.2% vs. 75.5%), and NPV (44.7% vs. 38.2%). When assessed by AUC, MRI performed the best, while CAD had the lowest. AI-CAD showed no significant difference from mammography, ultrasound, or DWI, suggesting that it performs comparably to conventional methods but surpasses CAD.

In the univariate analysis conducted to identify significant predictors of pCR, several imaging modalities, including AI-CAD, mammography, ultrasound, and MRI, were significant, with the exception of CAD and DWI. Pathologic factors such as histologic grade, as well as ER, PR, HER2, and Ki-67 status, also showed statistical significance. In the subsequent multivariable analysis, negative MRI findings, histologic grade 3, and HER2-positive status emerged as significant predictors of pCR. It is well established that breast MRI is the most accurate imaging modality for assessing tumor response during or after NAC [5,6,7]. Although there is some variation in the significant factors reported across different studies, HER2-positive status and high tumor grade are consistently recognized as factors that increase the likelihood of achieving pCR [41,42,43,44].

This study faced several limitations. Firstly, as a retrospective study with a relatively small sample size from a single institution, this limits generalizability. This constraint prevented subgroup analysis by molecular subtype or chemotherapy regimen. Future studies with larger sample sizes and subgroup analyses could uncover new insights into the capabilities and limitations of AI-CAD in evaluating treatment response. Secondly, we defined a negative exam as an image measurement of 0. While clinically aware that post-NAC mammography may show residual calcifications even in pCR cases, we aimed to maintain objectivity in image-based assessments. Therefore, any remaining calcifications were measured and classified as a positive exam, which may have influenced our results. Subgroup analysis considering specific mammographic features, such as the presence of calcifications, was not feasible in this study but may be valuable in future research. Lastly, only one type of AI-CAD software and a single vendor’s mammography system were used in our analysis. The results may vary with different vendors or AI-CAD systems. Future studies incorporating various AI-CAD systems and mammography vendors could provide a more comprehensive understanding of AI-CAD’s effectiveness and generalizability across platforms.

In conclusion, AI-CAD did not reach the performance of MRI in assessing treatment response but showed improved specificity compared to conventional CAD and performed comparably to ultrasound and DWI, demonstrating its potential as a tool for predicting treatment response in breast cancer patients.

## Figures and Tables

**Table 1:** Clinical, imaging, and pathological characteristics of the study population.

	Total (*n* = 132)	pCR (*n* = 37)	non-pCR (*n* = 95)	*p*-Value
Age (years)	51.6 ± 10.2	52.2 ± 9.0	51.3 ± 10.6	0.64
Breast density				0.64
Almost entirely fatty	6 (4.5)	2 (5.4)	4 (4.2)	
Scattered	21 (15.9)	5 (13.5)	16 (16.8)	
Heterogeneously dense	68 (51.5)	22 (59.5)	46 (48.4)	
Extremely dense	37 (28.0)	8 (21.6)	29 (30.5)	
Image findings, pre-NAC				
Mammography				0.87
Mass	64 (48.5)	19 (51.4)	45 (47.4)	
Calcification	3 (2.3)	1 (2.7)	2 (2.1)	
Mass with calcification	65 (49.2)	17 (45.9)	48 (50.5)	
Ultrasound				0.71
Mass	96 (72.7)	25 (67.6)	71 (74.7)	
Nonmmass	24 (18.2)	8 (21.6)	16 (16.8)	
Mass with nonmass	12 (9.1)	4 (10.8)	8 (8.4)	
MRI				0.33
Mass	80 (60.6)	20 (54.1)	60 (63.2)	
Nonmmass	27 (20.5)	7 (18.9)	20 (21.1)	
Mass with nonmass	25 (18.9)	10 (27.0)	15 (15.8)	
MRI distribution				0.15
Single	79 (59.8)	17 (45.9)	62 (65.3)	
Multifocal	26 (19.7)	12 (32.4)	14 (14.7)	
Multicentric	10 (7.6)	3 (8.1)	7 (7.4)	
Diffuse, inflammatory	16 (12.1)	5 (13.5)	11 (11.6)	
Bilateral	1 (0.8)	0 (0.0)	1 (1.1)	
Image measurement				
Mammography size (mm)				
Pre-NAC	43.4 ± 21.1	44.1 ± 24.4	43.1 ± 19.9	0.96
Post-NAC	25.2 ± 24.8	23.2 ± 31.2	26.0 ± 22.0	0.07
Difference	18.1 ± 15.9	20.9 ± 16.4	17.0 ± 15.7	0.18
Ultrasonography size (mm)				
Pre-NAC	37.5 ± 15.8	36.4 ± 16.7	38.0 ± 15.5	0.45
Post-NAC	17.0 ± 16.1	12.7 ± 18.6	18.7 ± 14.8	0.006
Difference	20.5 ± 14.1	23.7 ± 12.6	19.3 ± 14.6	0.03
MRI size (mm)				
Pre-NAC	48.6 ± 22.4	50.0 ± 24.8	48.1 ± 21.5	0.87
Post-NAC	17.7 ± 21.7	6.7 ± 18.3	22.0 ± 21.5	<0.001
Difference	30.9 ± 22.6	43.3 ± 25.6	26.0 ± 19.4	<0.001
DWI (×10^−6^/mm^3^)				
Mean ADC value, pre-NAC	787 ± 150	821 ± 173	774 ± 139	0.18
Mean ADC value, post-NAC	985 ± 267	-	985 ± 267	N/A
Histologic grade				0.001
Grade 1	5 (3.8)	0 (0.0)	5 (5.3)	
Grade 2	76 (57.6)	14 (37.8)	62 (65.3)	
Grade 3	51 (38.6)	23 (62.2)	28 (29.5)	
Lymph node involvement				0.047
Negative	86 (65.2)	29 (78.4)	57 (60.0)	
Positive	46 (34.8)	8 (21.6)	38 (40.0)	
Estrogen receptor				0.006
Negative	64 (48.5)	25 (67.6)	39 (41.1)	
Positive	68 (51.5)	12 (32.4)	56 (58.9)	
Progesterone receptor				0.01
Negative	81 (61.4)	29 (78.4)	52 (54.7)	
Positive	51 (38.6)	8 (21.6)	43 (45.3)	
HER2				<0.001
Negative	67 (50.8)	7 (18.9)	60 (63.2)	
Positive	65 (49.2)	30 (81.1)	35 (36.8)	
Ki-67				0.02
<20%	46 (34.8)	7 (18.9)	39 (41.1)	
≥20%	86 (65.2)	30 (81.1)	56 (58.9)	
IHC type				<0.001
Luminal A	19 (14.4)	1 (2.7)	18 (18.9)	
Luminal B	52 (39.4)	12 (32.4)	40 (42.1)	
HER2-enriched	30 (22.7)	20 (54.1)	10 (10.5)	
Triple-negative	31 (23.5)	4 (10.8)	27 (28.4)	
Operation title				0.15
Breast-conserving surgery	115 (87.1)	35 (94.6)	80 (84.2)	
Mastectomy	17 (12.9)	2 (5.4)	15 (15.8)	
Residual tumor size (mm)	18.0 ± 21.9	-	25.0 ± 22.1	<0.001
Residual invasive cancer size (mm)	11.6 ± 18.5	-	16.1 ± 20.0	<0.001
Residual DCIS size (mm)	12.2 ± 20.0	-	17.0 ± 21.9	<0.001

Continuous variables are displayed as the mean ± SD, and categorical variables are displayed as the number (percentage). *p*-values were derived using the chi-square test, Fisher’s exact test, Wilcoxon rank-sum test, or Student’s *t*-test. pCR: pathologic complete response; NAC: neoadjuvant chemotherapy; DWI: diffusion-weighted imaging; ADC: apparent diffusion coefficient; HER2: human epidermal growth factor receptor 2; IHC: immunohistochemical; DCIS: ductal carcinoma in situ; N/A: not applicable. Difference: difference between pre-NAC and post-NAC.

**Table 2:** Concordance between mammography, AI-CAD, and CAD according to mammography findings.

	Mammography Findings	Concordance Rate % (95% CI)		AI-CAD Abnormality Score (Median, [Range])
		CAD	AI-CAD	
pre-NAC	Mass with calcifications (*n* = 65)	100.0	96.9 (92.7, 100)	98 [94, 99]
	Mass (*n* = 64)	81.3 (71.7, 90.8)	96.9 (92.6, 100)	90 [62.75, 97]
	Calcifications (*n* = 3)	100.0	100	99 [68, 99]
	Total (*n* = 132)	90.9 (84.8, 94.7)	97.0 (94.1, 99.9)	96 [77.25. 99]
post-NAC	Mass with calcifications (*n* = 42)	95.2 (83.8, 99.4)	95.2 (83.8, 99.4)	91 [57.5, 96]
	Negative (*n* = 33)	78.8 (61.1, 91.0)	100.0	0
	Mass (*n* = 32)	81.3 (63.6, 92.8)	81.3 (63.6, 92.8)	45.5 [14.5, 87.5]
	Calcifications (*n* = 25)	100.0	76.0 (54.9, 90.6)	81 [11, 95]
	Total (*n* = 132)	88.6 (82.0, 93.5)	89.4 (82.8, 94.1)	42.5 [0, 91]

pCR: pathologic complete response; NAC: neoadjuvant chemotherapy; AI-CAD: artificial intelligence-based computer-aided detection.

**Table 3:** Diagnostic performance of imaging modalities in post-NAC.

	Sensitivity (95% CI)	Specificity (95% CI)	PPV (95% CI)	NPV (95% CI)	AUC (95% CI)
CAD	77.9 (68.2, 85.8)	35.1 (20.2, 52.5)	75.5 (65.8, 83.6)	38.2 (22.2, 56.4)	0.565 (0.476, 0.651)
AI-CAD	72.6 (62.5, 81.3)	56.8 (39.5, 72.9)	81.2 (71.2, 88.8)	44.7 (30.2, 59.9)	0.647 (0.559, 0.728)
Mammography	83.2 (74.1, 90.1)	45.9 (29.5, 63.1)	79.8 (70.5, 87.2)	51.5 (33.5, 69.2)	0.646 (0.558, 0.727)
Ultrasound	86.3 (77.7, 92.5)	43.2 (27.1, 60.5)	79.6 (70.5, 86.9)	55.2 (35.7, 73.6)	0.648 (0.560, 0.729)
MRI	76.8 (67.1, 84.9)	78.4 (61.8, 90.2)	90.1 (81.5, 95.6)	56.9 (42.2, 70.7)	0.776 (0.695, 0.844)
DWI	46.3 (36.3, 56.3)	100.0 (90.4, 100.0)	100.0 (92.0, 100.0)	42.1 (31.7, 52.4)	0.732 (0.648, 0.805)

pCR: pathologic complete response; NAC: neoadjuvant chemotherapy; AI-CAD: artificial intelligence-based computer-aided detection; DWI: diffusion-weighted imaging; PPV: positive predictive value; NPV: negative predictive value; CI: confidence interval.

**Table 4:** Association between pCR and imaging modalities in post-NAC.

	pCR (*n* = 37)	Non-pCR (*n* = 95)	*p*-Value
CAD			0.124
Negative	13 (35.1)	21 (22.1)	
Positive	24 (64.9)	74 (77.9)	
AI-CAD			0.002
Negative	21 (56.8)	26 (27.4)	
Positive	16 (43.2)	69 (72.6)	
AI-CAD abnormality score	0 [0, 77]	59 [0, 92]	0.001
Mammography			0.001
Negative	17 (45.9)	16 (16.8)	
Positive	20 (54.1)	79 (83.2)	
Ultrasound			<0.001
Negative	16 (43.2)	13 (13.7)	
Positive	21 (56.8)	82 (86.3)	
MRI			<0.001
Negative	29 (78.4)	22 (23.2)	
Positive	8 (21.6)	73 (76.8)	
DWI			<0.001
Negative	37 (100.0)	51 (53.7)	
Positive	0 (0.0)	44 (46.3)	

Values are represented as number (percentage) or median [interquartile range]. *p*-values were derived using the chi-square test, Fisher’s exact test, Wilcoxon rank-sum test, or Student’s *t*-test. pCR: pathologic complete response; NAC: neoadjuvant chemotherapy; AI-CAD: artificial intelligence-based computer-aided detection; DWI: diffusion-weighted imaging; CI: confidence interval.

**Table 5:** Univariate and multivariable logistic regression analysis for pCR prediction.

	Univariate Analysis		Multivariable Analysis	
	Odds Ratio (95% CI)	*p*-Value	Adjusted Odds Ratio (95% CI)	*p*-Value
Age (years)		0.36		
<50	reference			
≥50	1.45 (0.65, 3.23)			
Breast density		0.65		
Almost entirely fatty	reference			
Scattered	0.63 (0.09, 4.49)	0.64		
Heterogeneously dense	0.96 (0.16, 5.63)	0.96		
Extremely dense	0.55 (0.08, 3.58)	0.53		
CAD		0.13		
Positive	reference			
Negative	1.91 (0.83, 4.38)			
AI-CAD		0.002		0.65
Positive	reference		reference	
Negative	3.48 (1.58, 7.69)		1.56 (0.23, 10.7)	
Mammography		0.001		0.35
Positive	reference		reference	
Negative	4.20 (1.81, 9.73)		2.49 (0.37, 16.9)	
Ultrasound		<0.001		0.99
Positive	reference		reference	
Negative	4.81 (2.00, 11.5)		0.99 (0.24, 4.18)	
MRI		<0.001		<0.001
Positive	reference		reference	
Negative	12.0 (4.81, 30.1)		12.6 (3.28, 48.5)	
DWI		0.997		
Positive	reference			
Negative	(0, ∞)			
Histologic grade		0.01		0.001
Grades 1 and 2	reference		reference	
Grade 3	3.93 (1.77, 3.73)		9.03 (2.40, 33.9)	
Lymph node involvement		0.05		
Negative	reference			
Positive	0.41 (0.17, 1.00)			
Estrogen receptor		0.007		0.56
Negative	reference		reference	
Positive	0.33 (0.15, 0.74)		0.64 (0.14, 2.92)	
Progesterone receptor		0.015		0.62
Negative	reference		reference	
Positive	0.33 (0.14, 0.81)		0.67 (0.14, 3.27)	
HER2		<0.001		<0.001
Negative	reference		reference	
Positive	7.35 (2.92, 18.5)		14.68 (3.42, 62.9)	
Ki-67		0.02		0.09
<20%	reference		reference	
≥20%	2.99 (1.19, 7.48)		3.50 (0.82, 14.9)	

pCR: pathologic complete response; CI: confidence interval; AI-CAD: artificial intelligence-based computer-aided detection; NAC: neoadjuvant chemotherapy; DWI: diffusion-weighted imaging; HER2: human epidermal growth factor receptor 2.

## Data Availability

The datasets used and/or analyzed in the current study are available from the corresponding author upon reasonable request.

## References

[1] Cho N., Park I.-A., Kwon B.R., Shin S.U., Kim S.Y., Lee S.H., Chang J.M., Moon W.K. (2018). Dynamic contrast-enhanced breast MRI for evaluating residual tumor size after neoadjuvant chemotherapy. Radiology.

[2] King T.A., Morrow M. (2015). Surgical issues in patients with breast cancer receiving neoadjuvant chemotherapy. Nat. Rev. Clin. Oncol..

[3] Cortazar P., Zhang L., Untch M., Mehta K., Costantino J.P., Wolmark N., Bonnefoi H., Cameron D., Gianni L., Valagussa P. (2014). Pathological complete response and long-term clinical benefit in breast cancer: The CTNeoBC pooled analysis. Lancet.

[4] Jeevan R., Cromwell D.A., Trivella M., Lawrence G., Kearins O., Pereira J., Sheppard C., Caddy C.M., van der Meulen J.H.P. (2012). Reoperation rates after breast conserving surgery for breast cancer among women in England: Retrospective study of hospital episode statistics. BMJ.

[5] Lobbes M., Prevos R., Smidt M., Tjan-Heijnen V., Van Goethem M., Schipper R., Beets-Tan R.G., Wildberger J.E. (2013). The role of magnetic resonance imaging in assessing residual disease and pathologic complete response in breast cancer patients receiving neoadjuvant chemotherapy: A systematic review. Insights Imaging.

[6] Marinovich M.L., Houssami N., Macaskill P., Sardanelli F., Irwig L., Mamounas E.P., Von Minckwitz G., Brennan M.E., Ciatto S. (2013). Meta-analysis of magnetic resonance imaging in detecting residual breast cancer after neoadjuvant therapy. J. Natl. Cancer Inst..

[7] Yuan Y., Chen X.S., Liu S.Y., Shen K.W. (2010). Accuracy of MRI in prediction of pathologic complete remission in breast cancer after preoperative therapy: A meta-analysis. AJR Am. J. Roentgenol..

[8] Ko E.S., Han B.-K., Kim R.B., Ko E.Y., Shin J.H., Hahn S.Y., Nam S.J., Lee J.E., Lee S.K., Im Y.-H. (2013). Analysis of factors that influence the accuracy of magnetic resonance imaging for predicting response after neoadjuvant chemotherapy in locally advanced breast cancer. Ann. Surg. Oncol..

[9] Chen J., Bahri S., Mehta R.S., Carpenter P.M., McLaren C.E., Chen W., Fwu P.T., Hsiang D.J.B., Lane K.T., Butler J.A. (2014). Impact of factors affecting the residual tumor size diagnosed by MRI following neoadjuvant chemotherapy in comparison to pathology. J. Surg. Oncol..

[10] Bouzón A., Acea B., Soler R., Iglesias Á., Santiago P., Mosquera J., Calvo L., Seoane-Pillado T., García A. (2016). Diagnostic accuracy of MRI to evaluate tumour response and residual tumour size after neoadjuvant chemotherapy in breast cancer patients. Radiol. Oncol..

[11] Chu W., Jin W., Liu D., Wang J., Geng C., Chen L., Huang X. (2018). Diffusion-weighted imaging in identifying breast cancer pathological response to neoadjuvant chemotherapy: A meta-analysis. Oncotarget.

[12] Bray F., Ferlay J., Soerjomataram I., Siegel R., Torre L., Jemal A. (2020). Global cancer statistics 2018: GLOBOCAN estimates of incidence and mortality worldwide for 36 cancers in 185 countries. CA Cancer J. Clin..

[13] Kerlikowske K., Grady D., Rubin S.M., Sandrock C., Ernster V.L. (1995). Efficacy of screening mammography: A meta-analysis. JAMA.

[14] Ko J.M., Nicholas M.J., Mendel J.B., Slanetz P.J. (2006). Prospective assessment of computer-aided detection in interpretation of screening mammography. AJR Am. J. Roentgenol..

[15] Taplin S.H., Rutter C.M., Lehman C.D. (2006). Testing the effect of computer-assisted detection on interpretive performance in screening mammography. AJR Am. J. Roentgenol..

[16] Henriksen E.L., Carlsen J.F., Vejborg I.M., Nielsen M.B., Lauridsen C.A. (2019). The efficacy of using computer-aided detection (CAD) for detection of breast cancer in mammography screening: A systematic review. Acta Radiol..

[17] Salim M., Wåhlin E., Dembrower K., Azavedo E., Foukakis T., Liu Y., Smith K., Eklund M., Strand F. (2020). External evaluation of 3 commercial artificial intelligence algorithms for independent assessment of screening mammograms. JAMA Oncol..

[18] Rodriguez-Ruiz A., Lång K., Gubern-Merida A., Broeders M., Gennaro G., Clauser P., Helbich T.H., Chevalier M., Tan T., Mertelmeier T. (2019). Stand-alone artificial intelligence for breast cancer detection in mammography: Comparison with 101 radiologists. Radiology.

[19] Dembrower K., Wåhlin E., Liu Y., Salim M., Smith K., Lindholm P., Eklund M., Strand F. (2020). Effect of artificial intelligence-based triaging of breast cancer screening mammograms on cancer detection and radiologist workload: A retrospective simulation study. Lancet Digit. Health.

[20] Kim H.E., Kim H.H., Han B.K., Kim K.H., Han K., Nam H., Lee E.H., Kim E.K. (2020). Changes in cancer detection and false-positive recall in mammography using artificial intelligence: A retrospective, multireader study. Lancet Digit. Health..

[21] McKinney S.M., Sieniek M., Godbole V., Godwin J., Antropova N., Ashrafian H., Back T., Chesus M., Corrado G.S., Darzi A. (2020). International evaluation of an AI system for breast cancer screening. Nature.

[22] Yoon J.H., Kim E.K. (2021). Deep learning-based artificial intelligence for mammography. Korean J. Radiol..

[23] Kühl J., Elhakim M.T., Stougaard S.W., Rasmussen B.S.B., Nielsen M., Gerke O., Larsen L.B., Graumann O. (2024). Population-wide evaluation of artificial intelligence and radiologist assessment of screening mammograms. Eur. Radiol..

[24] Lee J.H., Kim K.H., Lee E.H., Ahn J.S., Ryu J.K., Park Y.M., Shin G.W., Kim Y.J., Choi H.Y. (2022). Improving the performance of radiologists using artificial intelligence-based detection support software for mammography: A multi-reader study. Korean J. Radiol..

[25] Kwon M.-R., Chang Y., Ham S.-Y., Cho Y., Kim E.Y., Kang J., Park E.K., Kim K.H., Kim M., Kim T.S. (2024). Screening mammography performance according to breast density: A comparison between radiologists versus standalone intelligence detection. Breast Cancer Res..

[26] Elhakim M.T., Stougaard S.W., Graumann O., Nielsen M., Gerke O., Larsen L.B., Rasmussen B.S.B. (2024). AI-integrated screening to replace double reading of mammograms: A population-wide accuracy and feasibility study. Radiol. Artif. Intell..

[27] Dembrower K., Crippa A., Colón E., Eklund M., Strand F., ScreenTrustCAD Trial Consortium (2023). Artificial intelligence for breast cancer detection in screening mammography in Sweden: A prospective, population-based, paired-reader, non-inferiority study. Lancet Digit. Health.

[28] Hickman S.E., Payne N.R., Black R.T., Huang Y., Priest A.N., Hudson S., Kasmai B., Juette A., Nanaa M., Aniq M.I. (2023). Mammography breast cancer screening triage using deep learning: A UK retrospective study. Radiology.

[29] Gjesvik J., Moshina N., Lee C.I., Miglioretti D.L., Hofvind S. (2024). Artificial intelligence algorithm for subclinical breast cancer detection. JAMA Netw. Open.

[30] Lee S.E., Kim G.R., Yoon J.H., Han K., Son W.J., Shin H.J., Moon H.J. (2023). Artificial intelligence assistance for women who had spot compression view: Reducing recall rates for digital mammography. Acta Radiol..

[31] Kim Y.S., Jang M.J., Lee S.H., Kim S.Y., Ha S.M., Kwon B.R., Moon W.K., Chang J.M. (2022). Use of artificial intelligence for reducing unnecessary recalls at screening mammography: A simulation study. Korean J. Radiol..

[32] Do Y.A., Jang M., Yun B., Shin S.U., Kim B., Kim S.M. (2021). Diagnostic performance of artificial intelligence-based computer-aided diagnosis for breast microcalcification on mammography. Diagnostics.

[33] Yoon J., Lee H.S., Kim M.J., Park V.Y., Kim E.K., Yoon J.H. (2022). AI-CAD for differentiating lesions presenting as calcifications only on mammography: Outcome analysis incorporating the ACR BI-RADS descriptors for calcifications. Eur. Radiol..

[34] Lee S.H., Shin H.J., Moon W.K. (2021). Diffusion-Weighted Magnetic Resonance Imaging of the Breast: Standardization of Image Acquisition and Interpretation. Korean J. Radiol..

[35] D’Orsi C., Bassett L., Feig S. (2018). Breast imaging reporting and data system (BI-RADS). Breast Imaging Atlas.

[36] Skarping I., Larsson M., Förnvik D. (2022). Analysis of mammograms using artificial intelligence to predict response to neoadjuvant chemotherapy in breast cancer patients: Proof of concept. Eur. Radiol..

[37] Wang-Lopez Q., Chalabi N., Abrial C., Radosevic-Robin N., Durando X., Mouret-Reynier M.A., Benmammar K.E., Kullab S., Bahadoor M., Chollet P. (2015). Can pathologic complete response (pCR) be used as a surrogate marker of survival after neoadjuvant therapy for breast cancer?. Crit. Rev. Oncol./Hematol..

[38] Kaufmann M., von Minckwitz G., Mamounas E.P., Cameron D., Carey L.A., Cristofanilli M., Denkert C., Eiermann W., Gnant M., Harris J.R. (2012). Recommendations from an international consensus conference on the current status and future of neoadjuvant systemic therapy in primary breast cancer. Ann. Surg. Oncol..

[39] Park C.C., Mitsumori M., Nixon A., Recht A., Connolly J., Gelman R., Silver B., Hetelekidis S., Abner A., Harris J.R. (2000). Outcome at 8 years after breast-conserving surgery and radiation therapy for invasive breast cancer: Influence of margin status and systemic therapy on local recurrence. J. Clin. Oncol..

[40] von Minckwitz G., Untch M., Blohmer J.-U., Costa S.D., Eidtmann H., Fasching P.A., Gerber B., Eiermann W., Hilfrich J., Huober J. (2012). Definition and impact of pathologic complete response on prognosis after neoadjuvant chemotherapy in various intrinsic breast cancer subtypes. J. Clin. Oncol..

[41] Janssen L., den Dekker B., Gilhuijs K., van Diest P., van der Wall E., Elias S. (2022). MRI to assess response after neoadjuvant chemotherapy in breast cancer subtypes: A systematic review and meta-analysis. NPJ Breast Cancer.

[42] Kim K.I., Lee K.H., Kim T.R., Chun Y.S., Lee T.H., Park H.K. (2014). Ki-67 as a predictor of response to neoadjuvant chemotherapy in breast cancer patients. J. Breast Cancer.

[43] Choi M., Park Y.H., Ahn J.S., Im Y.-H., Nam S.J., Cho S.Y., Cho E.Y. (2017). Evaluation of pathologic complete response in breast cancer patients treated with neoadjuvant chemotherapy: Experience in a single institution over a 10-year period. J. Pathol. Transl. Med..

[44] Goorts B., van Nijnatten T.J., de Munck L., Moossdorff M., Heuts E.M., de Boer M., Lobbes M.B., Smidt M.L. (2017). Clinical tumor stage is the most important predictor of pathological complete response rate after neoadjuvant chemotherapy in breast cancer patients. Breast Cancer Res. Treat..

